# (2*S*)-2-[(2S*,5R*,6R*)-5,6-Dimeth­oxy-5,6-dimethyl-1,4-dioxan-2-yl]-1-[(*S*)-1,1-dimethyl­ethylsulfon­yl]aziridine

**DOI:** 10.1107/S1600536810048816

**Published:** 2010-11-27

**Authors:** Toni Moragas Solà, William Lewis, Sampada V. Bettigeri, Robert A. Stockman, David C. Forbes

**Affiliations:** a School of Chemistry, University of Nottingham, Nottingham NG7 2RD, England; b Department of Chemistry, University of South Alabama, Mobile, AL 36688-0002, USA

## Abstract

The reaction of a sulfur ylide with a chiral non-racemic sulfinyl imine afforded the desired aziridine in excellent yield and subsequent oxidation of the sulfinyl moiety dissolved in anhydrous dichloro­methane using a 75% aqueous solution of 3-chloro­per­oxy­benzoic acid afforded the title compound, C_14_H_27_NO_6_S. The configuration of the newly formed stereogenic center at the point of attachment of the 1,4-dioxane ring to the aziridine ring is *S*. The configurations of the pre-existing sites 2-, 5-, and 6-positions of the 1,4-dioxane ring prior to reaction of sulfinyl imine with the sulfur ylide are *S*, *R*, and *R*, respectively. The C—N bond lengths of the aziridine are 1.478 (2) and 1.486 (2) Å.

## Related literature

For the first synthesis of the title compound, see: Forbes *et al.* (2009 ▶). For the use of sulfinyl imines in the preparation of aziridines, see: Forbes *et al.* (2009 ▶); Chigboh *et al.* (2008 ▶); Morton *et al.* (2006 ▶). For a review on the use sulfur ylide technologies in the preparation of three-membered rings, see: McGarrigle *et al.* (2007 ▶). For the use of *tert*-butyl sulfinyl groups as stereodiscriminating groups, see: Ellman *et al.* (2002 ▶); Wakayama & Ellman (2009 ▶). For the use of three-carbon building blocks in the assembly of systems of medicinal significance, specifically HIV protease inhibitors, see: Izawa & Onishi (2006 ▶); Honda *et al.* (2004 ▶).
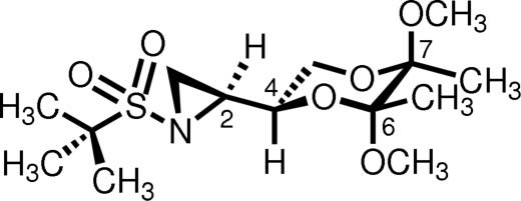


## Experimental

### 

#### Crystal data

C_14_H_27_NO_6_S
*M* *_r_* = 337.43
Monoclinic, 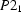

*a* = 8.31483 (9) Å
*b* = 10.31672 (10) Å
*c* = 10.33015 (11) Å
β = 91.0961 (10)°
*V* = 885.98 (2) Å^3^
*Z* = 2
Cu *K*α radiation
μ = 1.86 mm^−1^
*T* = 90 K
0.95 × 0.67 × 0.15 mm

#### Data collection

Oxford Diffraction SuperNova, single source at offset, Atlas diffractometer
Absorption correction: analytical [*CrysAlis PRO* (Oxford Diffraction, 2010) ▶; analytical numeric absorption correction using a multifaceted crystal model (Clark & Reid, 1995 ▶)] *T*_min_ = 0.320, *T*_max_ = 0.764
48647 measured reflections
3548 independent reflections
3532 reflections with *I* > 2σ(*I*)
*R*_int_ = 0.082

#### Refinement

*R*[*F* ^2^ > 2σ(*F* ^2^)] = 0.038
*wR*(*F* ^2^) = 0.104
*S* = 1.10
3548 reflections
206 parameters
1 restraint
H-atom parameters constrained
Δρ_max_ = 0.25 e Å^−3^
Δρ_min_ = −0.36 e Å^−3^
Absolute structure: Flack (1983 ▶), 1653 Friedel pairs
Flack parameter: −0.009 (13)

### 

Data collection: *CrysAlis PRO* (Oxford Diffraction, 2010 ▶); cell refinement: *CrysAlis PRO*; data reduction: *CrysAlis PRO*; program(s) used to solve structure: *SHELXS97* (Sheldrick, 2008 ▶); program(s) used to refine structure: *SHELXL97* (Sheldrick, 2008 ▶); molecular graphics: *OLEX2* (Dolomanov *et al.*, 2009 ▶); software used to prepare material for publication: *publCIF* (Westrip, 2010 ▶).

## Supplementary Material

Crystal structure: contains datablocks I, global. DOI: 10.1107/S1600536810048816/hb5738sup1.cif

Structure factors: contains datablocks I. DOI: 10.1107/S1600536810048816/hb5738Isup2.hkl

Additional supplementary materials:  crystallographic information; 3D view; checkCIF report

## Figures and Tables

**Fig. 1.**
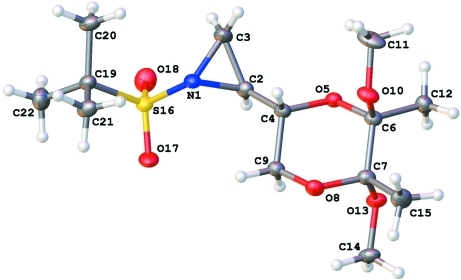
The molecular structure of 1 showing 50% displacement ellipsoids.

## supplementary crystallographic information

### Comment

Chiral non-racemic three-carbon building blocks are common intermediates used in the assembly of many HIV protease inhibitors as demonstrated by Honda *et al.* (2004) and Izawa & Onishi (2006). Working with not epoxide but aziridine functionality offers the synthetic organic chemist a viable alternative approach toward the advancement of these materials of biological and medicinal importance as reported by Chigboh *et al.* (2008), Ellman *et al.* (2002), Morton *et al.* (2006), McGarrigle *et al.* (2007), and Wakayama & Ellman (2009). As terminal aziridines can be readily obtained using sulfur ylide technologies from the corresponding imines, both enantiomeric lines can be prepared when starting with D-mannitol and ascorbic acid and the properly juxtaposed chiral non-racemic sulfinyl imine. Proof of concept was first published by Forbes *et al.* (2009). That is, reaction of methylphenylsulfonium methylide with both enantiomeric lines of the butanediacetal-protected chiral non-racemic sulfinyl imines resulted in dastereomeric ratios of >95:5. The sulfur ylide methylphenylsulfonium methylide was generated *in situ* upon thermal decarboxylation of carboxylmethyl betaine functionality. Alternatively using trimethylsulfonium iodide in dimethylsulfoxide in the presence of base, the sulfur ylide generated by this route, dimethylsulfonium methylide, reacted as well with the sulfinyl imine [S(*S*), *N*(*E*)]-2-methyl-*N*-[((2*S*,5*R*,6*R*)-5,6-dimethoxy-5,6-dimethyl-1,4-dioxacyclohexyl)methylene]-2-propanesulfinamide to afford as major isomer the title compound upon oxidation of the sulfinyl aziridine. This was confirmed by NMR analysis of the products obtained using dimethylsulfonium methylide and methylphenylsulfonium methylide with both diastereomeric lines of sulfinyl imine ([S(*S*), *N*(*E*)]-2-methyl-*N*-[((2*S*,5*R*,6*R*)-5,6-dimethoxy-5,6-dimethyl-1,4-dioxacyclohexyl)methylene]-2-propanesulfinamide and [S(*R*), *N*(*E*)]-2-methyl-*N*-[((2*S*,5*R*,6*R*)-5,6-dimethoxy-5,6-dimethyl-1,4-dioxacyclohexyl)methylene]-2-propanesulfinamide). Missing is the configuration of the newly formed center of the aziridine upon methylene transfer at C2. While attempts to grow crystals suitable for X-ray analysis of the sulfinyl aziridine itself and derivatives such as the deprotected aziridine were unsuccessful, success was obtained upon oxidation of the sulfinyl aziridine using *m*-chloroperoxybenzoic acid. The title compound, C_14_H_27_NO_6_S, was isolated in excellent yield and offered definitive evidence of the newly formed aziridine center (C2) as S. The configurations of the preexisting sites C4, C6, and C7 prior to reaction of sulfinyl imine with sulfur ylide are S, *R*, and *R*, respectively. The configuration of The C—N bond lengths of the aziridine are 1.478 (2) and 1.486 (2) Å.

### Experimental

(2*S*)-1-[*S*(*S**)-(1,1-dimethylethyl)sulfinyl]-2-[(2*S**,5*R**, 6*R**)-2-(5,6-dimethoxy- 5,6-dimethyl-1,4-dioxacyclohexyl)]aziridine

To a 60% solution of sodium hydride (203 mg, 5.03 mmol) in anhydrous dimethylsulfoxide (6 ml) was added trimethylsulfonium iodide (1.025 g, 5.03 mmol). This was stirred until the cloudy mixture went clear. At this point a solution of ([S(*S*), *N*(*E*)]-2-methyl-*N*-[((2*S*,5*R*,6*R*)-5,6-dimethoxy-5,6-dimethyl-1,4-dioxacyclohexyl) methylene]-2-propanesulfinamide (515 mg, 0.167 mmol) in anhydrous dimethylsulfoxide (4 ml) was added dropwise to the mixture and the solution was stirred at room temperature for 30 minutes. Once complete, ice-cold brine (5 ml) was added, and the reaction stirred for 5 minutes. The resulting mixture was filtered through a pad of Celite, and the solution extracted with ethyl acetate (3x5 ml), and concentrated under reduced pressure. The residue was partitioned between 1:1 petroleum ether/ethyl acetate and water, and the organic fraction dried over anhydrous sodium sulfate. Purification by column chromatography over silica gel (eluting with 5:1 petroleum ether/ethyl acetate) afforded the title compound (108 mg, 20% yield). [α]_D_ -94 (c 1/2, CHCl_3_); ν_max_ (CHCl_3_)/cm^-1^ 3010, 2835, 1521, 1475, 1425, 1377, 1192, 1142, 1078; δ_H_ (CDCl_3_, 300 MHz) 4.03 (1*H*, dt, *J* 11.2 and 3.2), 3.70 (1*H*, t, *J* 11.2), 3.48 (1*H*, dd, *J* 11.2 and 3.2), 3.24 (6*H*, s), 2.69 (1*H*, m), 2.17 (1*H*, d, *J* 4.2), 2.01 (1*H*, d, *J* 7.1), 1.27 (3*H*, s), 1.25 (3*H*, s), 1.24 (9*H*, s); δ_c_ (75 MHz, CDCl_3_) 99.2, 98.2, 65.2, 61.1, 56.8, 48.0, 29.9, 24.9, 22.6, 17.5; *m/z* (ESI+) 344 (*M*+23, 100%), 322 (*M*+1, 4); HRMS calculated for [C_14_H_28_NO_5_S]^+^ (*M*+Na^+^) 322.1683, found 322.1686.

(2*S*)-1-[*S*-(1,1-dimethylethyl)sulfonyl]-2-[(2*S**,5*R**,6*R**)-2-(5,6-dimethoxy-5,6- dimethyl-1,4-dioxacyclohexyl)]aziridine

To a solution of(2*S*)-1-[*S*-(*S**)-(1,1-dimethylethyl)sulfinyl]-2-[(2*S**,5*R**,6*R**)-2-(5,6-dimethoxy -5,6-dimethyl-1,4-dioxacyclohexyl)]aziridine (47 mg, 0.146 mmol) in anhydrous dichloromethane (1.5 ml) was added a 75% solution of *m*-chloroperoxybenzoic acid in water (34 mg, 0.148 mmol) and the mixture was stirred for five minutes. A saturated aqueous solution of sodium bicarbonate (2 ml) was added and the product was extracted with dichloromethane (2 ml) and washed with brine (2 × 1 ml). The organic layers were dried over anhydrous sodium sulfate and concentrated *in vacuo* to afford the title compound (47 mg, 95% yield). Recrystallization with ethyl ether/petroleum ether afforded the title compound as white crystals. m.p. = 90–95 °C; [α]_D_ -136 (c 1/2, CHCl_3_); ν_max_ (CHCl_3_)/cm^-1^ 3011, 1522, 1477, 1424, 1193, 1034; δ_H_ (CDCl_3_, 300 MHz) 3.74–3.60 (2*H*, m), 3.47 (1*H*, d, *J* 9.5), 3.20 (3*H*, s), 3.18 (3*H*, s), 2.68 (1*H*, dd, *J* 6.9 and 4.4), 2.55 (1*H*, d, *J* 6.9), 2.20 (1*H*, d, *J* 4.4), 1.41 (9*H*, s), 1.21 (6*H*, s); δ_c_ (75 MHz, CDCl_3_) 99.2, 98.2, 67.4, 60.9, 59.6, 48.0, 36.7, 31.8, 24.1, 17.6; *m/z* (ESI+) 360 (*M*+23, 100%), 338 (*M*+1, 7); HRMS calculated for [C_14_H_27_NNaO_6_S]^+^ (*M*+Na^+^) 360.1451, found 360.1460.

### Refinement

All H atoms were placed in calculated positions and allowed to ride during subsequent refinement with *U*_iso_(H) = 1.5*U*_eq_(C) and C—H distances of 0.98 Å for the methyl H atoms, *U*_iso_(H) = 1.2*U*_eq_(C) and C—H distances of 0.99 Å for the methylene H atoms, and *U*_iso_(H) = 1.2*U*_eq_(C) and C—H distances of 1.00 Å for the methine H atoms.

### Crystal data

**Table:**

C_14_H_27_NO_6_S	*F*(000) = 364
*M**_r_* = 337.43	*D*_x_ = 1.265 Mg m^−^^3^
Monoclinic, *P*2_1_	Cu *K*α radiation, λ = 1.5418 Å
Hall symbol: P 2yb	Cell parameters from 44949 reflections
*a* = 8.31483 (9) Å	θ = 4.3–73.3°
*b* = 10.31672 (10) Å	µ = 1.86 mm^−^^1^
*c* = 10.33015 (11) Å	*T* = 90 K
β = 91.0961 (10)°	Slab, colourless
*V* = 885.98 (2) Å^3^	0.95 × 0.67 × 0.15 mm
*Z* = 2	

### Data collection

**Table:**

Oxford Diffraction SuperNova, single source at offset, Atlas diffractometer	3548 independent reflections
Radiation source: SuperNova (Cu) X-ray Source	3532 reflections with *I* > 2σ(*I*)
mirror	*R*_int_ = 0.082
Detector resolution: 10.3613 pixels mm^-1^	θ_max_ = 73.4°, θ_min_ = 4.3°
ω scans	*h* = −10→10
Absorption correction: analytical [*CrysAlis PRO* (Oxford Diffraction, 2010); analytical numeric absorption correction using a multifaceted crystal model (Clark & Reid, 1995)]	*k* = −12→12
*T*_min_ = 0.320, *T*_max_ = 0.764	*l* = −12→12
48647 measured reflections	

### Refinement

**Table:**

Refinement on *F*^2^	Secondary atom site location: difference Fourier map
Least-squares matrix: full	Hydrogen site location: inferred from neighbouring sites
*R*[*F*^2^ > 2σ(*F*^2^)] = 0.038	H-atom parameters constrained
*wR*(*F*^2^) = 0.104	*w* = 1/[σ^2^(*F*_o_^2^) + (0.0774*P*)^2^ + 0.1298*P*] where *P* = (*F*_o_^2^ + 2*F*_c_^2^)/3
*S* = 1.10	(Δ/σ)_max_ < 0.001
3548 reflections	Δρ_max_ = 0.25 e Å^−^^3^
206 parameters	Δρ_min_ = −0.35 e Å^−^^3^
1 restraint	Absolute structure: Flack (1983), 1653 Friedel pairs
Primary atom site location: structure-invariant direct methods	Flack parameter: −0.009 (13)

### Special details

**Table:**

Geometry. All e.s.d.'s (except the e.s.d. in the dihedral angle between two l.s. planes) are estimated using the full covariance matrix. The cell e.s.d.'s are taken into account individually in the estimation of e.s.d.'s in distances, angles and torsion angles; correlations between e.s.d.'s in cell parameters are only used when they are defined by crystal symmetry. An approximate (isotropic) treatment of cell e.s.d.'s is used for estimating e.s.d.'s involving l.s. planes.
Refinement. Refinement of *F*^2^ against ALL reflections. The weighted *R*-factor *wR* and goodness of fit *S* are based on *F*^2^, conventional *R*-factors *R* are based on *F*, with *F* set to zero for negative *F*^2^. The threshold expression of *F*^2^ > 2σ(*F*^2^) is used only for calculating *R*-factors(gt) *etc*. and is not relevant to the choice of reflections for refinement. *R*-factors based on *F*^2^ are statistically about twice as large as those based on *F*, and *R*- factors based on ALL data will be even larger.

### Fractional atomic coordinates and isotropic or equivalent isotropic displacement parameters (Å^2^)

**Table:**

	*x*	*y*	*z*	*U*_iso_*/*U*_eq_	
N1	0.37564 (17)	0.58231 (13)	0.58518 (14)	0.0196 (3)	
C2	0.5315 (2)	0.51956 (18)	0.61615 (16)	0.0205 (3)	
H2	0.5588	0.4412	0.5639	0.025*	
C3	0.5278 (2)	0.6453 (2)	0.54586 (19)	0.0284 (4)	
H3A	0.5631	0.7238	0.5937	0.034*	
H3B	0.5539	0.6448	0.4528	0.034*	
C4	0.56425 (18)	0.51094 (17)	0.75921 (15)	0.0186 (3)	
H4	0.5312	0.5935	0.8019	0.022*	
O5	0.73405 (13)	0.49225 (12)	0.77640 (10)	0.0181 (2)	
C6	0.7813 (2)	0.48353 (17)	0.90943 (15)	0.0190 (3)	
C7	0.6875 (2)	0.37256 (17)	0.97603 (16)	0.0199 (3)	
O8	0.51910 (14)	0.38873 (12)	0.95304 (11)	0.0205 (3)	
C9	0.4763 (2)	0.39781 (17)	0.81885 (16)	0.0198 (3)	
H9A	0.3587	0.4105	0.8087	0.024*	
H9B	0.5055	0.3165	0.7741	0.024*	
O10	0.73723 (16)	0.59700 (13)	0.97601 (13)	0.0243 (3)	
C11	0.8058 (3)	0.7144 (2)	0.9290 (2)	0.0368 (5)	
H11B	0.7977	0.7160	0.8342	0.055*	
H11C	0.9192	0.7192	0.9562	0.055*	
H11A	0.7476	0.7887	0.9643	0.055*	
C12	0.9618 (2)	0.46183 (19)	0.90905 (17)	0.0238 (4)	
H12A	1.0041	0.4623	0.9983	0.036*	
H12C	1.0131	0.5312	0.8598	0.036*	
H12B	0.9850	0.3780	0.8689	0.036*	
O13	0.74372 (15)	0.25843 (12)	0.91606 (12)	0.0219 (3)	
C14	0.6670 (2)	0.14069 (18)	0.95495 (18)	0.0261 (4)	
H14C	0.6912	0.0718	0.8930	0.039*	
H14A	0.5504	0.1541	0.9572	0.039*	
H14B	0.7068	0.1157	1.0413	0.039*	
C15	0.7090 (2)	0.3703 (2)	1.12238 (17)	0.0293 (4)	
H15A	0.6786	0.4547	1.1580	0.044*	
H15C	0.8219	0.3522	1.1449	0.044*	
H15B	0.6406	0.3026	1.1587	0.044*	
S16	0.26511 (4)	0.50297 (4)	0.47432 (3)	0.02020 (12)	
O17	0.21224 (16)	0.38671 (12)	0.53743 (14)	0.0278 (3)	
O18	0.35073 (16)	0.48822 (16)	0.35537 (13)	0.0323 (3)	
C19	0.09768 (19)	0.61192 (18)	0.45030 (16)	0.0205 (3)	
C20	0.1621 (2)	0.74360 (18)	0.40680 (19)	0.0267 (4)	
H20A	0.2301	0.7807	0.4759	0.040*	
H20C	0.0718	0.8021	0.3880	0.040*	
H20B	0.2256	0.7323	0.3287	0.040*	
C21	0.0075 (2)	0.62361 (19)	0.57736 (18)	0.0264 (4)	
H21B	−0.0386	0.5393	0.5998	0.040*	
H21C	−0.0790	0.6876	0.5673	0.040*	
H21A	0.0822	0.6515	0.6464	0.040*	
C22	−0.0086 (2)	0.5508 (2)	0.3442 (2)	0.0338 (4)	
H22B	0.0512	0.5465	0.2634	0.051*	
H22C	−0.1054	0.6037	0.3308	0.051*	
H22A	−0.0395	0.4631	0.3704	0.051*	

### Atomic displacement parameters (Å^2^)

**Table:**

	*U*^11^	*U*^22^	*U*^33^	*U*^12^	*U*^13^	*U*^23^
N1	0.0192 (7)	0.0145 (7)	0.0251 (7)	−0.0006 (5)	0.0015 (5)	0.0016 (5)
C2	0.0174 (7)	0.0205 (9)	0.0239 (8)	0.0015 (6)	0.0039 (6)	0.0021 (6)
C3	0.0226 (8)	0.0290 (9)	0.0335 (9)	−0.0073 (7)	0.0004 (7)	0.0119 (8)
C4	0.0168 (7)	0.0169 (8)	0.0221 (7)	−0.0004 (7)	0.0040 (5)	0.0012 (6)
O5	0.0171 (5)	0.0175 (5)	0.0199 (5)	−0.0001 (5)	0.0038 (4)	0.0014 (5)
C6	0.0200 (7)	0.0165 (8)	0.0207 (7)	−0.0008 (6)	0.0033 (6)	−0.0021 (6)
C7	0.0217 (9)	0.0182 (8)	0.0200 (7)	−0.0015 (7)	0.0041 (6)	0.0003 (6)
O8	0.0210 (6)	0.0214 (6)	0.0192 (5)	−0.0015 (5)	0.0055 (4)	0.0001 (4)
C9	0.0194 (8)	0.0183 (8)	0.0219 (7)	−0.0021 (6)	0.0022 (6)	0.0006 (6)
O10	0.0259 (6)	0.0176 (6)	0.0297 (7)	−0.0045 (5)	0.0081 (5)	−0.0067 (5)
C11	0.0378 (12)	0.0178 (9)	0.0553 (13)	−0.0092 (8)	0.0160 (10)	−0.0113 (9)
C12	0.0195 (8)	0.0272 (9)	0.0248 (8)	−0.0014 (6)	0.0018 (6)	0.0020 (7)
O13	0.0252 (6)	0.0164 (6)	0.0244 (6)	0.0008 (5)	0.0077 (5)	0.0020 (5)
C14	0.0285 (9)	0.0189 (8)	0.0312 (9)	−0.0007 (7)	0.0089 (7)	0.0066 (7)
C15	0.0326 (9)	0.0358 (10)	0.0196 (8)	−0.0043 (8)	0.0031 (7)	0.0015 (8)
S16	0.0213 (2)	0.0156 (2)	0.0238 (2)	0.00156 (15)	0.00127 (14)	−0.00083 (14)
O17	0.0297 (7)	0.0131 (6)	0.0404 (7)	−0.0024 (5)	−0.0035 (5)	0.0021 (5)
O18	0.0329 (7)	0.0361 (8)	0.0281 (6)	0.0092 (6)	0.0054 (5)	−0.0064 (6)
C19	0.0188 (8)	0.0192 (8)	0.0235 (8)	0.0012 (6)	0.0024 (6)	0.0046 (6)
C20	0.0278 (9)	0.0205 (8)	0.0321 (9)	0.0031 (7)	0.0087 (7)	0.0081 (7)
C21	0.0237 (8)	0.0248 (9)	0.0310 (9)	0.0049 (7)	0.0093 (7)	0.0073 (7)
C22	0.0289 (9)	0.0398 (11)	0.0323 (9)	−0.0014 (8)	−0.0068 (8)	0.0023 (9)

### Geometric parameters (Å, °)

**Table:**

N1—C2	1.478 (2)	C12—H12A	0.9800
N1—C3	1.486 (2)	C12—H12C	0.9800
N1—S16	1.6690 (14)	C12—H12B	0.9800
C2—C3	1.487 (3)	O13—C14	1.433 (2)
C2—C4	1.500 (2)	C14—H14C	0.9800
C2—H2	1.0000	C14—H14A	0.9800
C3—H3A	0.9900	C14—H14B	0.9800
C3—H3B	0.9900	C15—H15A	0.9800
C4—O5	1.4326 (18)	C15—H15C	0.9800
C4—C9	1.515 (2)	C15—H15B	0.9800
C4—H4	1.0000	S16—O17	1.4380 (14)
O5—C6	1.4248 (18)	S16—O18	1.4399 (14)
C6—O10	1.410 (2)	S16—C19	1.8027 (17)
C6—C12	1.518 (2)	C19—C21	1.529 (2)
C6—C7	1.553 (2)	C19—C22	1.531 (3)
C7—O13	1.414 (2)	C19—C20	1.531 (2)
C7—O8	1.425 (2)	C20—H20A	0.9800
C7—C15	1.519 (2)	C20—H20C	0.9800
O8—C9	1.428 (2)	C20—H20B	0.9800
C9—H9A	0.9900	C21—H21B	0.9800
C9—H9B	0.9900	C21—H21C	0.9800
O10—C11	1.428 (2)	C21—H21A	0.9800
C11—H11B	0.9800	C22—H22B	0.9800
C11—H11C	0.9800	C22—H22C	0.9800
C11—H11A	0.9800	C22—H22A	0.9800
			
C2—N1—C3	60.21 (11)	C6—C12—H12C	109.5
C2—N1—S16	113.74 (11)	H12A—C12—H12C	109.5
C3—N1—S16	119.18 (12)	C6—C12—H12B	109.5
N1—C2—C3	60.17 (11)	H12A—C12—H12B	109.5
N1—C2—C4	112.41 (14)	H12C—C12—H12B	109.5
C3—C2—C4	122.30 (16)	C7—O13—C14	115.48 (12)
N1—C2—H2	116.4	O13—C14—H14C	109.5
C3—C2—H2	116.4	O13—C14—H14A	109.5
C4—C2—H2	116.4	H14C—C14—H14A	109.5
N1—C3—C2	59.62 (11)	O13—C14—H14B	109.5
N1—C3—H3A	117.8	H14C—C14—H14B	109.5
C2—C3—H3A	117.8	H14A—C14—H14B	109.5
N1—C3—H3B	117.8	C7—C15—H15A	109.5
C2—C3—H3B	117.8	C7—C15—H15C	109.5
H3A—C3—H3B	114.9	H15A—C15—H15C	109.5
O5—C4—C2	106.87 (12)	C7—C15—H15B	109.5
O5—C4—C9	109.16 (13)	H15A—C15—H15B	109.5
C2—C4—C9	111.49 (13)	H15C—C15—H15B	109.5
O5—C4—H4	109.8	O17—S16—O18	117.33 (9)
C2—C4—H4	109.8	O17—S16—N1	105.49 (7)
C9—C4—H4	109.8	O18—S16—N1	111.26 (8)
C6—O5—C4	112.39 (11)	O17—S16—C19	109.91 (8)
O10—C6—O5	110.41 (13)	O18—S16—C19	109.97 (8)
O10—C6—C12	112.95 (14)	N1—S16—C19	101.67 (8)
O5—C6—C12	105.14 (13)	C21—C19—C22	111.19 (15)
O10—C6—C7	104.98 (13)	C21—C19—C20	111.23 (15)
O5—C6—C7	110.01 (13)	C22—C19—C20	110.84 (15)
C12—C6—C7	113.42 (14)	C21—C19—S16	108.74 (11)
O13—C7—O8	110.88 (13)	C22—C19—S16	105.99 (14)
O13—C7—C15	112.93 (15)	C20—C19—S16	108.67 (12)
O8—C7—C15	105.33 (13)	C19—C20—H20A	109.5
O13—C7—C6	104.27 (13)	C19—C20—H20C	109.5
O8—C7—C6	109.87 (14)	H20A—C20—H20C	109.5
C15—C7—C6	113.65 (15)	C19—C20—H20B	109.5
C7—O8—C9	113.28 (12)	H20A—C20—H20B	109.5
O8—C9—C4	109.40 (13)	H20C—C20—H20B	109.5
O8—C9—H9A	109.8	C19—C21—H21B	109.5
C4—C9—H9A	109.8	C19—C21—H21C	109.5
O8—C9—H9B	109.8	H21B—C21—H21C	109.5
C4—C9—H9B	109.8	C19—C21—H21A	109.5
H9A—C9—H9B	108.2	H21B—C21—H21A	109.5
C6—O10—C11	115.42 (13)	H21C—C21—H21A	109.5
O10—C11—H11B	109.5	C19—C22—H22B	109.5
O10—C11—H11C	109.5	C19—C22—H22C	109.5
H11B—C11—H11C	109.5	H22B—C22—H22C	109.5
O10—C11—H11A	109.5	C19—C22—H22A	109.5
H11B—C11—H11A	109.5	H22B—C22—H22A	109.5
H11C—C11—H11A	109.5	H22C—C22—H22A	109.5
C6—C12—H12A	109.5		
			
S16—N1—C2—C3	111.22 (14)	C6—C7—O8—C9	55.06 (17)
C3—N1—C2—C4	115.46 (17)	C7—O8—C9—C4	−58.42 (18)
S16—N1—C2—C4	−133.32 (13)	O5—C4—C9—O8	58.54 (17)
S16—N1—C3—C2	−102.22 (14)	C2—C4—C9—O8	176.38 (13)
C4—C2—C3—N1	−99.05 (17)	O5—C6—O10—C11	−59.72 (19)
N1—C2—C4—O5	−160.31 (14)	C12—C6—O10—C11	57.7 (2)
C3—C2—C4—O5	−92.39 (19)	C7—C6—O10—C11	−178.25 (16)
N1—C2—C4—C9	80.49 (18)	O8—C7—O13—C14	−57.86 (17)
C3—C2—C4—C9	148.40 (16)	C15—C7—O13—C14	60.08 (19)
C2—C4—O5—C6	179.52 (14)	C6—C7—O13—C14	−176.06 (14)
C9—C4—O5—C6	−59.78 (17)	C2—N1—S16—O17	70.35 (13)
C4—O5—C6—O10	−58.81 (16)	C3—N1—S16—O17	138.26 (13)
C4—O5—C6—C12	179.07 (14)	C2—N1—S16—O18	−57.90 (14)
C4—O5—C6—C7	56.60 (17)	C3—N1—S16—O18	10.01 (16)
O10—C6—C7—O13	−174.97 (12)	C2—N1—S16—C19	−174.94 (12)
O5—C6—C7—O13	66.24 (15)	C3—N1—S16—C19	−107.03 (14)
C12—C6—C7—O13	−51.20 (17)	O17—S16—C19—C21	48.33 (14)
O10—C6—C7—O8	66.15 (16)	O18—S16—C19—C21	178.96 (13)
O5—C6—C7—O8	−52.65 (17)	N1—S16—C19—C21	−63.07 (13)
C12—C6—C7—O8	−170.08 (13)	O17—S16—C19—C22	−71.29 (14)
O10—C6—C7—C15	−51.58 (18)	O18—S16—C19—C22	59.34 (14)
O5—C6—C7—C15	−170.38 (14)	N1—S16—C19—C22	177.31 (12)
C12—C6—C7—C15	72.19 (19)	O17—S16—C19—C20	169.54 (12)
O13—C7—O8—C9	−59.68 (17)	O18—S16—C19—C20	−59.83 (14)
C15—C7—O8—C9	177.84 (14)	N1—S16—C19—C20	58.14 (13)
